# Vagitus Uterinus, Report of Two Cases

**Published:** 1914-01

**Authors:** F. J. Giuffrida

**Affiliations:** Chief of Out-Patient Obstetrical Department of the Atlanta Medical School, Atlanta, Ga.


### VAGITUS UTERINUS.

### Report of Two Cases

By F. J. Giuffrida,
Chief of Out-Patient Obstetrical Department of the Atlanta Medical School,
Atlanta, Ga.

Peculiar terminology, this vagitus uterinus (a baby crying in utero), not so peculiar as the truthful occurrence itself. There has been many discussions among obstetricians along this line of hearing different sounds in utero. Judging from the discussion of hiccoughs on up to crying, etc., heard from the lips of our budding obstetricians lead me to think that we shall hear of a baby cooing in utero. Some men actually hoot at the idea when told that a baby had been heard crying in utero. I expect it has the same effect on them as when they tell others that they never get a lacerated perineum. We cannot all expect to see or hear the same things in life, something is obliged to escape us once in a while, no matter how alert we are. Baby-crying in utero does not happen often, in fact it is very rare. Sometimes they cannot be made to cry after delivery. I desire to submit the report of two cases of vagitus, after having read Dr. Telfair's article on the same subject, which was published in the New York Medical Journal, October 11, 1913. This stimulated me to give a brief report as follows:

Mrs. R———, age 21, primipara. History good. Pelvis normal. Well developed woman.

I was called to case May 19, 1912. On examination I found frank breach (L. S. A.). There was nearly full dilatation, Contractions were strong and regular, and the Membranes intact. The anterior hip was leading, and becoming tightly engaged in pelvis. After preparing the table, I decided to assist in bringing down breach; the membranes were ruptured before placing her on table. Delivery of the breach 'caused considerable trouble necessitating maneuvers in different ways, which allowed air to get into uterus. After passing tape over groin of the foetus, I liberated the breach, and then my assistant called my attention to the baby's crying. Of course we were surprised to hear it. It kept up crying louder and louder. After I got the breach down to cord, I pulled it down and it was pulsating nicely. It took me about twelve minutes or more to deliver the baby, working hard all the while. It was crying continuously at about half minute intervals, and sobbing too, it could be heard six feet away easily. There could be no mistake as to where it came from and what it was. The child was not asphyxiated. It cried good immediately after birth. The perineum was lacerated and repaired. The placenta was delivered manually.

Case 2.—I was called to the second patient by students. The woman was seven months pregnant, the membranes were found ruptured, the right foot was in vagina, the cevix was dilated about the size of a dollar, I finished dilating by the Edgar Barnes method and while delivering the child it cried faintly two or three times. We all heard it. I delivered an asphyxiated baby hurriedly. Resuscitated it, but it died later in the afternoon.

---

Editor, Journal-Record of Medicine,

Dear Sir:

I desire to call your attention to the Malaria Section of the National Drainage Congress.

This Section was organized during the Third National Drainage Congress held at St. Louis, April 10-12, 1913.

Our objects shall be to stimulate the study of the distribution, prevalence and economic importance of malaria, to conduct a compaign of publicity as far as our means will permit, and to devise ways and means to effect a permanent and efficient campaign against this grave disease.

The next meeting of the Congress will be held in Savannah, Ga., in 1914, the exact date to be announced later. At this meeting an extensive malaria program is contemplated.

Your membership and co-operation are earnestly desired. We want you to attend the next meeting and to contribute to the program or take part in the discussions. The membership fee in the Section is two dollars. Please have this letter read before your medical society.

Hoping to have you with us, I am

Cordially,

WM. H. DEADERICK, Secty.

